# Ruderman–Kittel–Kasuya–Yosida-type interfacial Dzyaloshinskii–Moriya interaction in heavy metal/ferromagnet heterostructures

**DOI:** 10.1038/s41467-021-23586-y

**Published:** 2021-06-02

**Authors:** Taehyun Kim, In Ho Cha, Yong Jin Kim, Gyu Won Kim, Andrey Stashkevich, Yves Roussigné, Mohamed Belmeguenai, Salim M. Chérif, Alexander S. Samardak, Young Keun Kim

**Affiliations:** 1 Department of Materials Science and Engineering, Korea University, Seoul, Korea; 2 Laboratoire des Sciences des Procédés et des Matériaux, CNRS-UPR 3407, Université Sorbonne Paris Nord, Villetaneuse, France; 3 School of Natural Sciences, Far Eastern Federal University, Vladivostok, Russia; 4 National Research South Ural State University, Chelyabinsk, Russia

**Keywords:** Ferromagnetism, Spintronics, Surfaces, interfaces and thin films

## Abstract

The manipulation of magnetization with interfacial modification using various spin-orbit coupling phenomena has been recently revisited due to its scientific and technological potential for next-generation memory devices. Herein, we experimentally and theoretically demonstrate the interfacial Dzyaloshinskii–Moriya interaction characteristics penetrating through a MgO dielectric layer inserted between the Pt and CoFeSiB. The inserted MgO layer seems to function as a chiral exchange interaction mediator of the interfacial Dzyaloshinskii–Moriya interaction from the heavy metal atoms to ferromagnet ones. The potential physical mechanism of the anti-symmetric exchange is based on the tunneling-like behavior of conduction electrons through the semi-conductor-like ultrathin MgO. Such behavior can be correlated with the oscillations of the indirect exchange coupling of the Ruderman–Kittel–Kasuya–Yosida type. From the theoretical demonstration, we could provide approximate estimation and show qualitative trends peculiar to the system under investigation.

## Abstract (web summary)

The mechanism of the interfacial Dzyaloshinskii-Moriya interaction in heavy metal-ferromagnet heterostructures is debated. Here, the authors show the oscillating behaviour of the interaction as a function of the MgO spacer layer thickness, supporting the interlayer exchange coupling mechanism of the Ruderman-Kittel-Kasuya-Yosida type.

## Introduction

Interface-induced phenomena have recently been revisited and experimentally illustrated because of their great potential to explore novel magnetic effects such as skyrmions, chiral domain wall dynamics, and spin-Hall effects^1–4^. Spin-orbit coupling (SOC), which is considered one of the most significant sources of the above-mentioned spin-related effects, creates a rapidly developing field of Spin-orbitronics and plays a crucial role in understanding the topology in magnetism^5^. Among various interfacial effects, the study of interfacial Dzyaloshinskii–Moriya interaction (iDMI) has attracted considerable attention due to its unique anti-symmetric exchange coupling behavior^6–8^.

The first prediction of the possibility of such unconventional exchange in systems with broken inversion symmetry was based on symmetry grounds, with no reference to any specific physical effect^9^. In low symmetry dielectrics, the combined effect of the SOC and exchange interaction can be simultaneously observed^10^. The prediction is essentially an extension of the super-exchange theory, including SOC, in which case the d–d orbital hybridization of core electrons plays the dominant role. However, a different mechanism was proposed for iDMI in metals^11^. In spin glasses with nonmagnetic heavy metal (HM) impurities, an additional term in the Ruderman–Kittel–Kasuya–Yosida (RKKY) interaction of the Dzyaloshinskii–Moriya type gives rise to the indirect exchange interaction. This interaction is due to the spin-orbit scattering of conduction electrons by the nonmagnetic impurities, which implies an active role of itinerant s electrons. In other words, an elementary iDMI cell referred to as a Fert’s triangle^12^ is considered as a triangle comprised of two magnetic atoms on the ferromagnet (FM) side linked to each other via a SOC-carrying atom on the HM side through the exchange mechanism. Importantly, in both mechanisms, the presence of SOC and symmetry reduction is instrumental^13^.

The physics behind the iDMI can be otherwise described. An alternative understanding regards the Rashba interfacial coupling^14^ as the critical mechanism behind iDMI. Notably, if the Rashba term is added to the Hamiltonian for the interface region’s electronic properties between the FM and nonmagnetic (NM) layers, chirality is introduced into the behavior of the electrons, which engenders iDMI^15^. The presence of the Rashba term predicts the generation of SOC-related effects, including iDMI, for a broader range of interfaces, including dielectric materials; oxides that do not necessarily contain heavy elements such as MgO/Si^16^ or FeCoB/TaO_x_^17^ have been experimentally confirmed.

Recently considerable theoretical efforts, including analytical formalisms and numerical ab initio calculations, aim to achieve a reliable description of the microscopic origin of the iDMI^7, 18, 19^. However, experimental investigation to unravel and thoroughly understand the underlying physics is still required. One method to test the physical nature of the bonds between the FM and HM atoms consists of inserting a spacer sub-layer separating the two constituent phases and controlling the distance separating the FM and HM atoms in the Fert’s triangle. The influence of a metallic spacer layer (Au and Ir) was experimentally investigated; the introduction of a monolayer (0.3–0.4 nm) leads to a remarkable threefold decline in interfacial iDMI strength, whereas a 1.2 nm one practically nullifies the iDMI strength^20^. Similarly, thin Cu spacer (~2 nm) leads to a similar threefold decline in iDMI strength^21^. All of the aforementioned cannot be easily reconciled with the exchange mechanism suggested^3^.

Herein, we report the results of the experimental investigation of the influence of a dielectric spacer with varying thicknesses on the strength of iDMI induced at the FM/HM interfaces supported by a toy model suggesting the potential physical mechanisms involved in conveying iDMI through a dielectric spacer.

## Results

### Magnetic properties and iDMI measurement

**Fig. 1:**
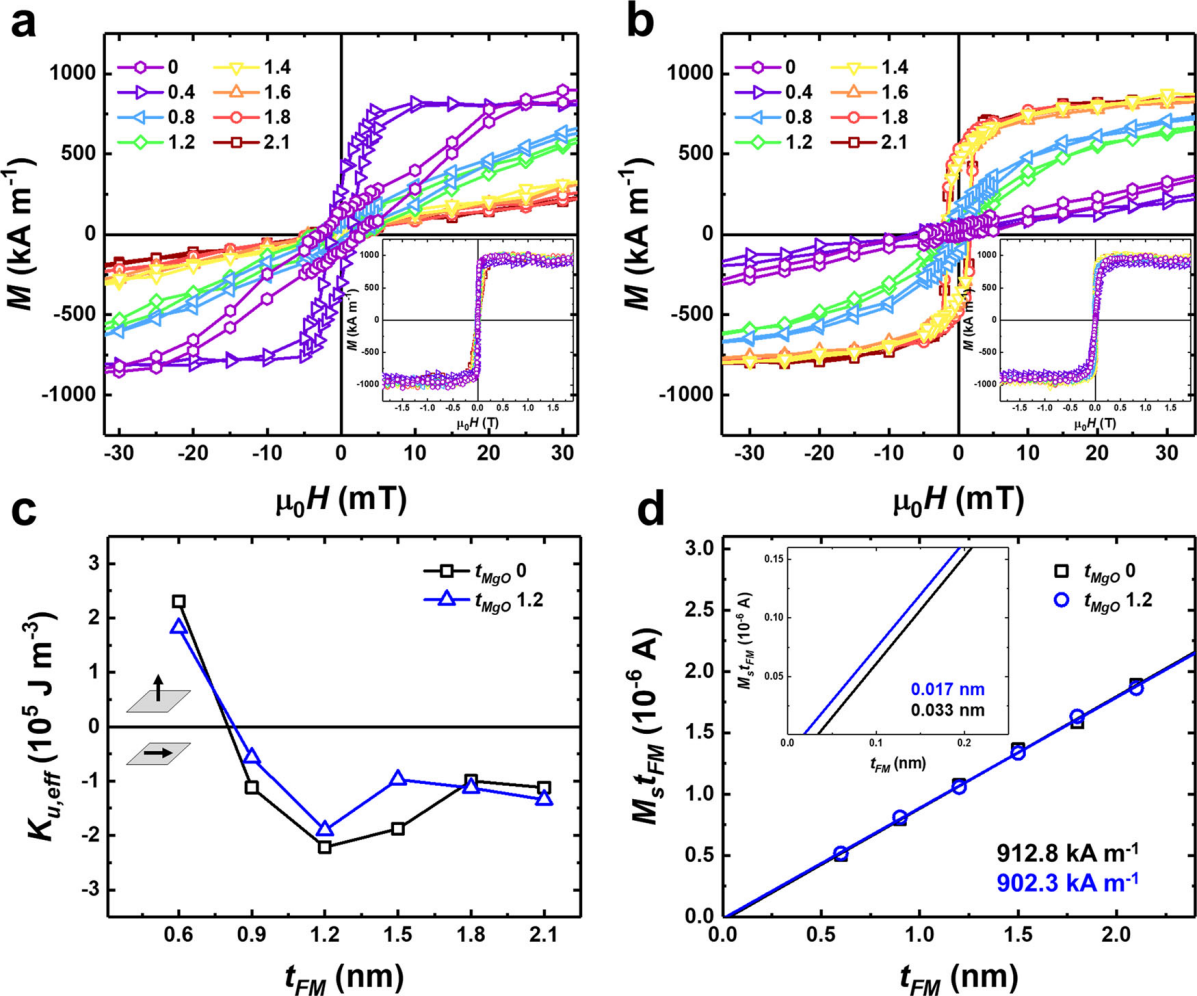
Magnetic properties of Ta(3)/Pt(5)/MgO(*t*_*MgO*_)/CoFeSiB(0.9)/MgO(1)/Ta(2). **a**, **b** The Out-of-plane (**a**), and in-plane (**b**) hysteresis loops for as-deposited samples. The inset shows full out of plane, and in-plane hysteresis loops. **c** Uniaxial anisotropy energy (*K*_*u,eff*_) as a function of CoFeSiB thickness (*t*_*FM*_). **d** Linear dependence of *M*_*s*_*t*_*FM*_ = f(*t*_*FM*_) exhibits effective saturation magnetization (*M*_*s*_^*eff*^) as a slope and magnetically dead layer as an *x*-intercept. *M*_*s*_^*eff*^ of each sample is depicted for each color, and a magnetically dead layer is written within the inset. Each color corresponds to the series of samples indicated in the legend.

We studied the structure of Ta(3)/Pt(5)/MgO(*t*_*MgO*_)/CoFeSiB(0.9)/MgO(1)/Ta(2). The thicknesses are written in nanometers, and *t*_*MgO*_ is varied from 0 to 2.1 nm. Due to the ultra-low deposition rate of MgO on the Pt layer, *t*_*MgO*_ could not be extended any further than 2.1 nm. Figure 1a, b depicts the out-of-plane and in-plane hysteresis loops for as-deposited samples with different *t*_*MgO*_ using vibrating sample magnetometry (VSM). The uniaxial anisotropy energy (*K*_*u,eff*_) of the samples was plotted as a function of the thickness of CoFeSiB for the samples with *t*_*MgO*_ = 0 and 1.2 nm, Fig. 1c. Volume and surface contribution to the magnetic anisotropy is analyzed using *K*_*eff*_*t*_*eff*_ and *t*_*FM,eff*_ plot. (Supplementary Note 1).

During the sputtering process, the intermixing and diffusion of atoms occurred, leading to the formation of a magnetically dead layer (MDL)^22^. The values of effective saturation magnetization (*M*_*s*_^*eff*^) and the MDL were measured using the samples with *t*_*MgO*_ of 0 and 1.2 nm. From the linear dependence of *M*_*s*_*t*_*FM*_ = f(*t*_*FM*_), where *M*_*s*_ was calculated as a fraction of the saturation magnetic moment (*M*) to a unit volume (*V*) of the CoFeSiB layer, the MDL thickness was deduced. The magnitudes of MDL thickness are 0.033 and 0.017 nm for the as-deposited sample when *t*_*MgO*_ is 0 and 1.2 nm, respectively (Fig. 1d). The *M*_*s*_^*eff*^ magnitudes of the as-deposited samples were 912.8 ± 17.5 and 902.3 ± 14.3 kA m^−1^ when *t*_*MgO*_ is 0 and 1.2 nm, respectively (Fig. 1d). The formation of the MDL was negligible, with low thickness; hence, there was no substantial difference in the value of *M*_*s*_^*eff*^.

The iDMI was measured using Brillouin light scattering (BLS) spectroscopy, which is the most direct and reliable measurement of the intrinsic iDMI constant proven so far^23^ (Supplementary Note 2). From the BLS spectra observed, the frequency difference between the Stokes and Anti-Stokes peaks (*Δf*) after the Lorentzian approximation can be deduced. Asymmetric pinning due to asymmetric surface perpendicular magnetic anisotropy (PMA) is an alternative mechanism leading to a non-reciprocity in spin-wave (SW) propagation indistinguishable from that induced by iDMI^24^. However, both contributions critically depend on the thickness of the ferromagnetic film *t*_*FM*_ in two completely different ways. While the PMA contribution tends to zero in ultrathin films, the iDMI induced non-reciprocity attains its highest possible values.

The PMA contribution stems from the fact that the Damon–Eshbach (DE) mode is inhomogeneous, confined to one of the interfaces depending on its propagation direction. In ultrathin FM films less than 4 nm, however, the transverse distribution of the dynamic magnetization in a DE SW mode becomes practically uniform for a given wavenumber^24^, and the PMA induced contribution becomes negligibly small (See Fig. 1 in Ref. ^24^).

We have undertaken ad hoc numerical simulations for the sample under study. More specifically, we have calculated the dispersion of two counter-propagating spin waves in an FM film in the presence of PMA in order to estimate the potential non-reciprocity of their propagation numerically. A detailed description of this kind of calculation can be found in the previous study by Roussigne et al*.*^25^. The SW non-reciprocity is parametrized by the frequency difference *Δf* between these counter-propagating waves for a given SW wave vector **k**_**sw**_ within the range covered by the BLS technique (0 < **k**_**sw**_ < 20 µm^−1^) due to the presence of PMA. In terms of Brillouin spectroscopy, it corresponds to the frequency difference between the Stokes and anti-Stokes peaks in BLS spectra appearing in Supplementary Eq. (1). As explained in the previous paragraph, the PMA induced non-reciprocity *Δf*_*PMA*_ becomes more pronounced for higher values of **k**_**sw**_. From symmetry considerations the SW non-reciprocity is only possible if it is induced by surface anisotropy and, importantly, if its distribution between two surfaces is asymmetric. The main purpose is to estimate numerically the maximum possible value of this spurious contribution to *Δf* to avoid misinterpretation of the experimental results. To ensure the reliability of this important numerical evaluation in our calculations, we have used the values of physical parameters based on their direct experimental estimates (VSM and BLS). While the overall effective PMA can be reliably extracted this way, its distribution between the two film surfaces cannot. To avoid underestimation of *Δf*_*PMA*_, our calculations were made under the assumption that PMA is localized entirely at one of the interfaces. They show that even in this case of maximum asymmetry, the non-reciprocity *Δf*_*PMA*_ of the DE mode in our ultrathin 0.9 nm CoFeSiB layer is on the order of 10 MHz. Thus the numerical estimation of the PMA induced non-reciprocity corresponds to ~1% with respect to the observed overall Stokes/anti-Stokes frequency asymmetry; PMA contribution is negligibly small. In other words, the observed effect of Stokes/anti-Stokes frequency asymmetry can be only due to the iDMI.

Figure 2a, b depicts two representative BLS spectra for the sample with the thickest MgO spacer layer (*t*_*MgO*_ = 2.1 nm) in positive and negative (±400 mT) fields. The phonon lines were perfectly symmetric, which means that the optical set-up is well-tuned. In contrast, the magnon spectral pattern demonstrates a pronounced asymmetry. Hence, the *D*_*eff*_ value was calculated and plotted as a function of the MgO spacer thickness (Fig. 2c). The main result was based on the fact that the MgO spacer remained practically transparent, even for thicknesses exceeding 2 nm.

**Figure:**
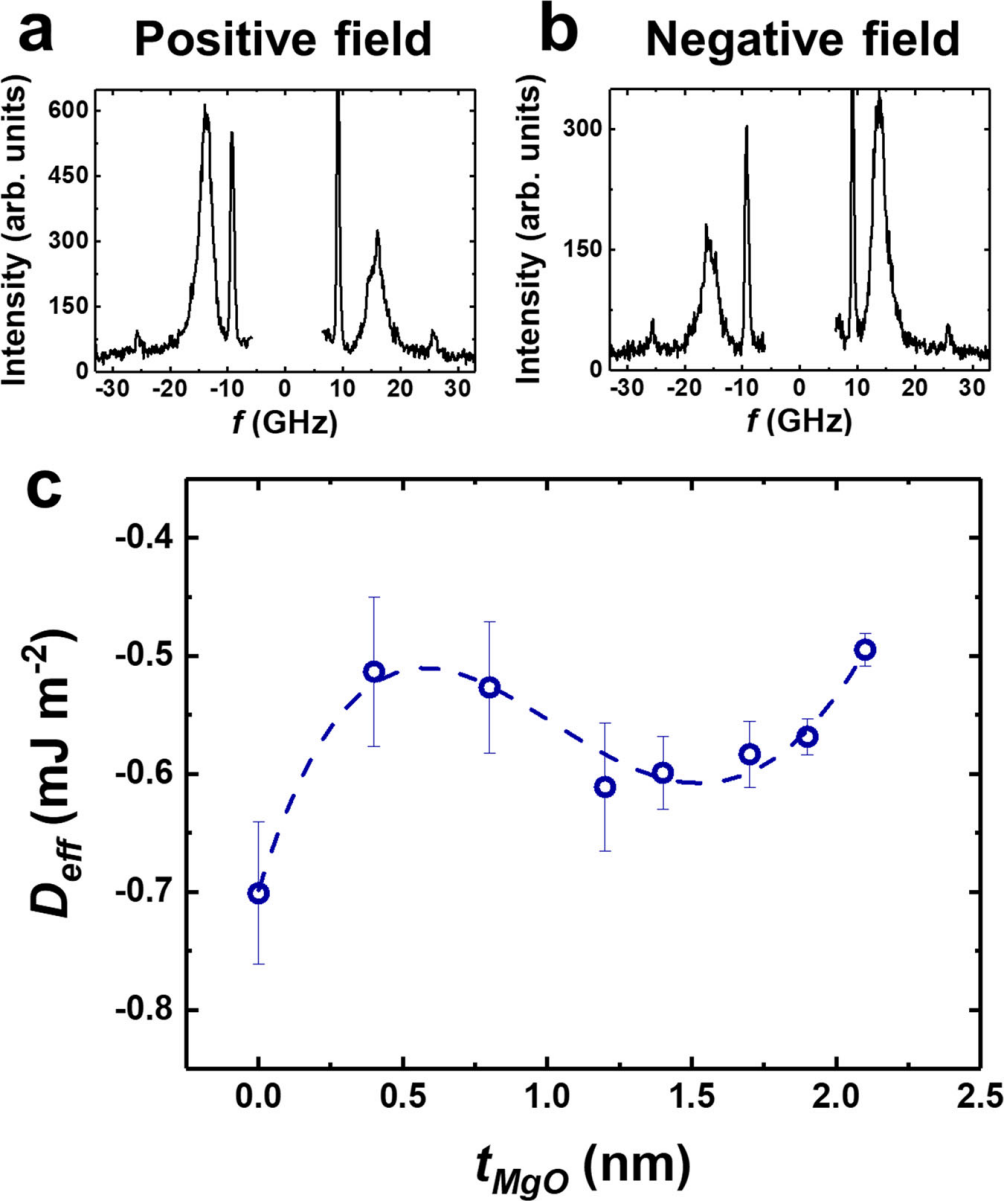
Fig. 2 Measured BLS spectra of Ta(3)/Pt(5)/MgO(*t*_*MgO*_)/CoFeSiB(0.9)/MgO(1)/Ta(2). **a**, **b** BLS spectrum signal recorded at **k**_**sw**_ = 22 µm^−1^ (*θ* = 60˚) with a positive (negative) saturating field of +400 mT (−400 mT) for a sample with *t*_*MgO*_ = 2.1 nm. **c** Effective iDMI energy density as a function of *t*_*MgO*_. Oscillating-like behavior of the iDMI energy density as a function of *t*_*MgO*_ for Ta(3)/Pt(5)/MgO(*t*_*MgO*_)/CoFeSiB(0.9)/MgO(1)/Ta(2). Error bars represent standard deviation, *n* = 2 independent replicates.

The addition of the MgO spacer layer between the HM and FM did not abruptly decrease the iDMI energy density; instead, it drops down slowly in a non-monotonic fashion resembling the oscillations in the case of RKKY coupling of two FM films through a nonmagnetic spacer until *t*_*MgO*_ reached 2.1 nm. Simultaneously, although the experimentally observed pattern is compatible with the suggested oscillatory description, we cannot insist categorically on this fact. This pattern can be seen as a single period of an oscillatory process or as a local maximum at *t*_*MgO*_ = 1.4 nm. Similarly, the minimum at 0.5 nm can also be seen as a single notch and not as systematic, regular oscillations. This interesting point will be addressed later in this manuscript in the section concerning our numerical simulation results.

The most pronounced Stokes/anti-Stokes frequency asymmetry (*Δf* = −2.38 ± 0.21 GHz) was observed in the structure without any MgO spacer. This corresponds to the iDMI constant value equivalent to *D*_*eff*_ = −0.70 ± 0.06 mJ m^−2^ and *D*_*s*_ = *D*_*eff*_ × *t*_*eff*_ = −0.61 ± 0.05 pJ m^−1^, which takes into account the effective FM thickness (*t*_*eff*_ = 0.867 nm). This result appeared reasonable and correlated with previously obtained results^8, 26^.

The negative value of *D*_*eff*_ supported the idea that the Pt layer was the primary source of iDMI, which is not surprising since the contribution of the Pt/CoFeSiB interface should be predominant. As *t*_*MgO*_ increased to 0.4 nm, a slow non-monotonic decrease of 30% was observed until *D*_*eff*_ = −0.51 ± 0.06 mJ m^−2^. Any further increase in *t*_*MgO*_ resulted in an increased iDMI constant up to *D*_*eff*_ = −0.61 ± 0.05 mJ m^−2^. This seeming oscillating behavior can be explained in terms of the tunneling of itinerant electrons through the MgO spacer linking the spin-carrying atoms (Co and Fe) on the FM side and the SOC-carrying atom on the HM side (Pt).

Theoretically, both interfaces involving Pt and MgO should be considered as potential contributors to the overall iDMI induced in the structure. Extensive ab initio numerical simulations^27, 28^ and experimental study^29^ concerning the alternative Rashba SOC mechanism typical for oxide/Co interfaces have shown that the contribution of the Co/MgO interface in Pt/Co/MgO multilayers can be rather pronounced caused by the interfacial oxidation after annealing^29^. However, in an experimental study on the effect of the Co/AlO_x_ separation interface with Cu, there was no contribution from the oxide layer to the iDMI constant^21^. The oxygen penetration enhanced the iDMI in some cases involving the annealing process after deposition; however, this was not observed in our case. In the publications mentioned earlier^27–29^, the structures are such that there is no way to independently estimate individual interfaces’ contributions (Pt/Co and Co/MgO). Furthermore, there were no available data on the direct measurements of MgO-induced iDMI at the interfaces between Co (or its alloys) and MgO.

Strictly speaking, one cannot exclude the contributions of the interfaces involving CoFeSiB/MgO. In the present study, a CoFeSiB FM film is sandwiched between two MgO layers. The fundamental symmetry of the iDMI is such that *DMI*_*MgO/CoFeSiB*_ and *DMI*_*CoFeSiB/MgO*_ have opposite signs. Hence, their contributions mutually cancel, *i.e*., ${|DM}{I}_{{MgO}/{CoFeSiB}/{MgO}}|={|DM}{I}_{{MgO}/{CoFeSiB}}|-{|DM}{I}_{{CoFeSiB}/{MgO}}|\approx 0$. Our theoretical toy formalism did not include the CoFeSiB/MgO interface effects but considering its contribution would improve the correlation between the experiment and theory (Figs. 2c and 4b).

### Material characterization of Pt and MgO

**Fig. 3:**
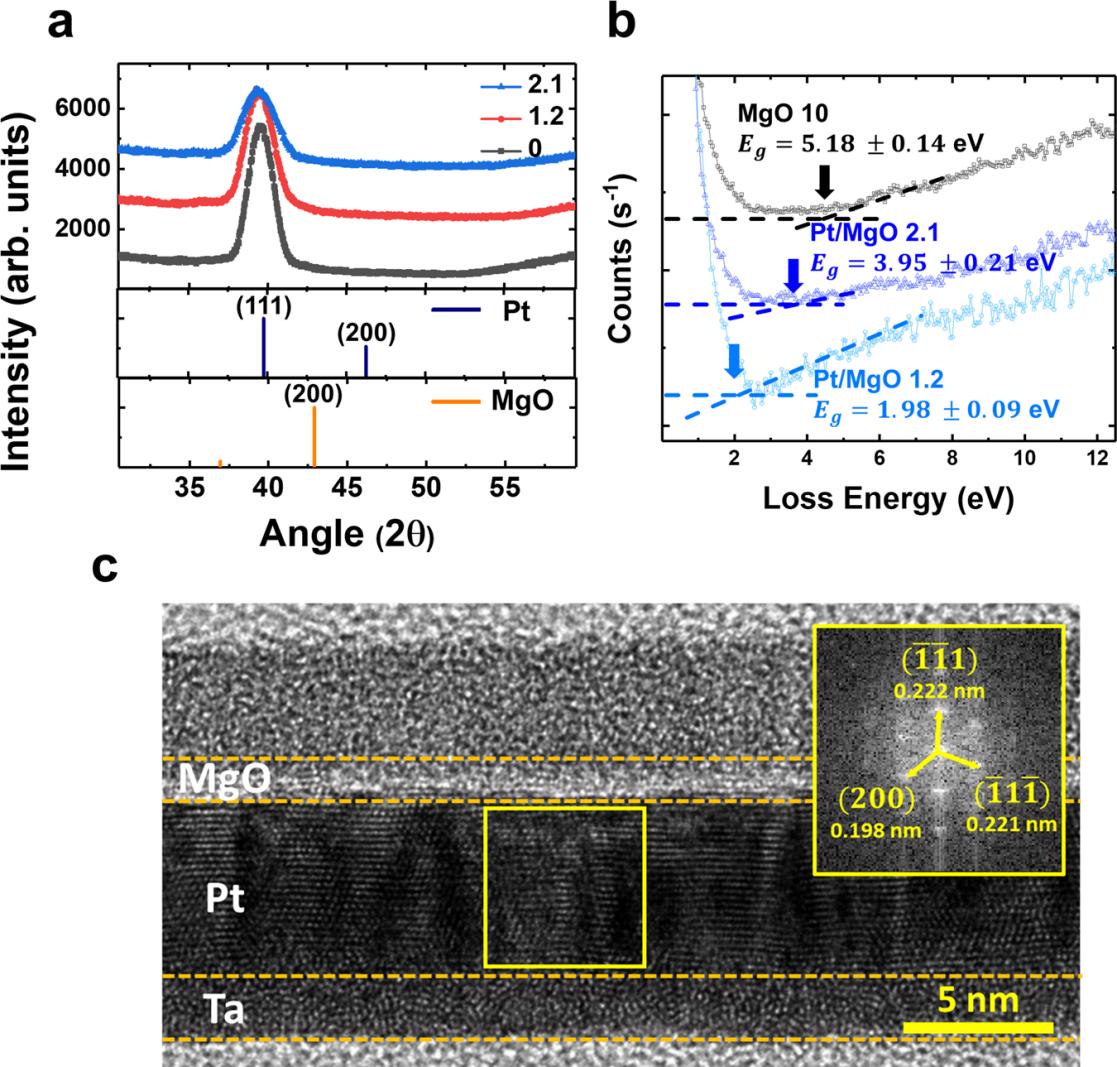
Microstructural analysis of the MgO inserted structure. **a** XRD spectra for the as-deposited Ta(3)/Pt(5)/MgO(*t*_*MgO*_)/CoFeSiB(0.9)/MgO(1)/Ta(2) films of various MgO thicknesses. The reference peak positions for Pt (ICDD No. 00-004-0802) and MgO (ICDD No. 00-0004-0829) are shown below the spectra by the vertical lines. **b** REELS spectra obtained for the as-deposited MgO film (black), Pt/MgO bilayer with MgO thickness of 1.2 nm (light blue), and 2.1 nm (dark blue) at the primary electron energy of 1 keV. **c** Cross-sectional HR-TEM image of the Ta(3)/Pt(5)/MgO(*t*_*MgO*_)/Ta sample with *t*_*MgO*_= 2.1 nm. The insets in the HR-TEM image consist of the 2D FFT patterns for the corresponding areas. The dashed line is inserted for eye guidance to separate the multilayer film stack.

Since the crystal structure of the Pt layer can affect the variation of iDMI magnitude^22^, microstructural analysis for Pt and MgO layers is conducted using X-ray diffraction (XRD) and high-resolution transmission electron microscope (HR-TEM). The XRD spectra shown in Fig. 3a exhibited no peaks corresponding to CoFeSiB, MgO, or Ta because the layers were ultrathin. All the samples with different *t*_*MgO*_ exhibited similar results, and fcc (111)-Pt exhibiting peaks at 39.77° is the dominant phase in the films deposited. The full width at half maxima (FWHM) of all the curves was smaller than 5°, demonstrating a good Pt (111) crystallinity of all the samples. The Pt peak was gradually shifted to a lower angle as *t*_*MgO*_ increased. We estimated the lattice parameter of the cubic crystal system by evaluating both Bragg’s law and the plane-spacing equation. The lattice parameters of Pt calculated at 0 and 2.1 nm of the MgO were 0.3937 and 0.3956 nm, respectively. This shift is due to the lattice mismatch between MgO and Pt during the growth of MgO. Given that fcc (111)-MgO with a peak position at 36.95° was deposited on top of the (111)-Pt surfaces, the existence of the MgO layer shifted the Pt peak to a lower angle.

A cross-sectional HR-TEM measurement was performed to verify the thickness and quality of a 2.1-nm-thick MgO spacer sample. From the HR-TEM image, the well-developed structure of Ta, Pt, and MgO was observed in Fig. 3c. The inset images of Fig. 3c correspond to the fast Fourier transform (FFT) diffraction pattern obtained by TEM, and the clear spot patterns instead of a vague ring pattern indicate that Pt has a crystalline structure. The atomic distance of deposited Pt agrees with the international center for diffraction data (ICDD), (PDF(Pt)#00-004-0802). The spot patterns observed from the TEM image coincide with the XRD results shown in Fig. 3a. Due to the oxidation of the top layers during the TEM sample preparation, only the crystal structure of Pt could be deduced. Scanning transmission electron spectroscopy (STEM) image with energy dispersive X-ray spectroscopy (EDX)-line profile and secondary ion mass spectroscopy (SIMS) elemental depth profile showing the full structure of the system under study are shown in Supplementary Note 4 and 5, respectively.

Our hypothesis stated that the MgO nonmagnetic spacer (NS), i.e., a material with a bulk bandgap energy (*E*_*g*_) of 7.8 eV^30^, behaves similarly to the effect of tunneling magnetoresistance (TMR), providing tunneling-like behavior of spin-polarized itinerant electrons between the HM and the FM. According to the Fert–Levy mechanism^11^, two magnetic atoms on the FM side are linked to each other via a SOC-carrying atom on the HM side by means of itinerant electrons. In the presence of a dielectric spacer, i.e., MgO, in our case, this is only possible if this spacer is sufficiently transparent. In other words, as soon as the MgO thickness exceeds the tunneling depth of the corresponding potential barrier, the HM layer will be cut off from the FM layer, and the iDMI will drop considerably, typically following an exponential pattern, which contradicts our experimental results (Fig. 2c).

Extensive research, both theoretical and experimental, conducted on TMR-related FM/MgO/FM trilayers within the last two decades^31–34^ revealed that the tunneling potential barrier’s height demonstrated considerable variation due to the high sensitivity to the technology involved in the sample preparation. The tunneling barrier height varied from 0.40 to 3.6 eV^35–37^, attributed to the oxygen vacancy defects in MgO. Consequently, there is a possibility that, in our case, the tunneling barrier may be very low, which can lead to a considerable increase in the tunneling depth. Moreover, the charge-neutral gap formed by the vacancies can even enable MgO to behave like a metal^38^, in which case the interlayer RKKY exchange constant demonstrates an oscillating-like behavior similar to that observed by us.

The bandgap was determined using reflection electron energy loss spectroscopy (REELS) because the photon-based bandgap energy measuring method can only probe significantly thicker layers^39, 40^. Figure 3b depicts different REELS spectra with primary energy of 1 keV for the different layers. The main elastic peak was aligned with the zero of the energy scales to examine the electronic excitations in each film; all the peaks were shifted vertically to indicate the bandgap energy of each sample. For a MgO single layer with a thickness of 10 nm, *E*_*g*_ was determined to be 5.187 ± 0.14 eV, whereas, in the case of the Pt/MgO bilayer with MgO thicknesses of 1.2 and 2.1 nm, *E*_*g*_ was determined to be 1.98 ± 0.09 eV and 3.95 ± 0.21 eV, respectively. The bandgap energy of the MgO single layer was close to the value of the surface band gap width of 6.3 eV reported earlier^40^. Oxygen vacancies mainly induce the reduction in the bandgap energy when MgO is adjacent to the metal layer. However, as the MgO thickness increased, the bandgap energy tended to increase as well. Therefore, in an ultra-thin MgO layer, the oxygen deficiency primarily caused the reduction in the bandgap energy of MgO, suggesting that the MgO spacer becomes more transparent for the itinerant electrons to tunnel through. However, this enhanced transparency alone cannot explain a very slight non-monotonic decrease in *D*_*eff*_ as a function of *t*_*MgO*_ observed experimentally. It should be mentioned that it bears a certain resemblance to the oscillating-like behavior of the interlayer RKKY exchange constant in the case of a conducting spacer. Since the quasi-conducting behavior of MgO spacers has been reported previously^38^, we have developed a simplified theoretical formalism taking advantage of this possibility.

### Nonmagnetic spacer effect on iDMI based on numerical simulation

Both indirect exchange coupling between two FM atoms through a tunnel barrier^38, 41, 42^, and the direct conventional Heisenberg exchange^43^ within an FM metal occur via the RKKY interaction. The conventional RKKY-related interlayer exchange coupling (IEC) typically describes the coupling between two FM layers separated by a nonmagnetic metallic spacer (Cu, Ag, Au)^44^. It could be extended to non-metallic insulating spacers by introducing the concept of a complex Fermi surface^45^. However, in our indirect iDMI mediated through a dielectric spacer, the RKKY coupling mechanism cannot be applied directly, thus requiring an ad hoc formalism developed along similar lines.

To qualitatively interpret our experimental findings on the non-trivial behavior of iDMI energy density, we propose a toy model, a very approximate calculation based on simple and easy-to-understand analytical expressions, providing more physical insight explaining untrivial fact in plain physical terms. The formalism stems from the approach developed by Bruno^44, 45^ to explain the significant features of the indirect inter-layer RKKY exchange coupling^45^.

Long-range magnetic interactions through nonmagnetic spacers of finite thickness have been experimentally observed^46–48^. The discussion around the physical mechanism behind the transparency of the corresponding spacers is not confined only to the long-range IDMI investigated in this paper. The previous study^48^ reported the oscillatory behavior of the dependence of the Gilbert damping on Cu spacer thickness (0 < *t*_*Cu*_ < 3 nm) in Py/Pt and Pt/Py structures with an assumption that coupling of spins at the Py/Cu and Cu/Pt interfaces through the Cu layer is analogous to the well-known RKKY interlayer exchange coupling between two FMs. At the same time, they acknowledge that the conventional RKKY model is not directly applicable, expressing hope that their results will stimulate the development of an adequate theoretical approach. In a sense, our toy theory of long-range iDMI outlined below can give a clue to understanding not only the long-range iDMI under study but also the mechanisms behind other SOC-related long-range interactions, which are of interest for spin-orbitronics.

The approach we take relies on the analysis of the influence of the energy terms describing a particular type of exchange interaction (conventional symmetric Heisenberg exchange or asymmetric DMI based exchange) on the overall Hamiltonian to identify energetically preferable electronic states. Such an approach leads to easily interpretable analytical formulae, providing for physical insight, which can be considered as its major advantage. At the same time, some of the basic assumptions are not self-evident, and an additional theoretical effort should be undertaken to justify them.

Major features of our RKKY-type formalism can be formulated as follows. In both cases of Bruno’s^44, 45^ and this study, a trilayer with a different structure is studied theoretically. We investigate a characteristically asymmetric FM/nonmagnetic spacer (NS)/HM trilayer, which differs appreciably from the conventional symmetric FM/NS/FM trilayer featuring the indirect RKKY inter-layer coupling.

Both approaches are based on the analysis of the following two primary parameters, namely, laws of quantization of the momenta of the quasi-free itinerant electrons dominating the physical properties of the system and their state of spin polarization as key factors determining the energy level of the investigated system. Thus, like P. Bruno, we assume that itinerant electron states in each constituent layer labeled by *n* with energy *ħω* and momentum $\vec{{{\bf{p}}}_{{\bf{n}}}}$ can be described by a plane wave with a wave vector $\vec{{{\bf{k}}}_{{\bf{n}}}}=\vec{{{\bf{p}}}_{{\bf{n}}}}/\hslash$^44^,

1 $$\varPsi ={\varPsi }^{n}{{\exp }}\big(i({k}_{1}{x}_{1}+{k}_{2}{x}_{2}+{k}_{3,n}{x}_{3}-\omega t)\big)$$

In a trilayer, *n* = 1, 2, 3 corresponds to Pt, MgO, and CoFeSiB, respectively. Each constituent layer *n* can be associated with a box with identical lateral dimensions *L*_*1*_ × *L*_*2*_ and specific thicknesses *L*_*3,n*_ (transverse dimension). Since *L*_*1*_ ≫ *L*_*3,n*_ and *L*_*2*_ ≫ *L*_*3,n*_ quantization along the lateral directions is negligible, which means that the in-plane components *k*_*1*_ and *k*_*2*_ (*p*_*1*_ and *p*_*2*_) form a continuum. It is the quantization of the transverse component *k*_*3,n*_ which plays a decisive role in our analysis, as is the case in the conventional theory of the RKKY inter-layer coupling. The quantization of the perpendicular components *k*_*3,n*_ is regulated by a continuity of the wave function at each interface:

2 $${\varPsi }^{n}{{\exp }}\left(i{k}_{3,n}{x}_{3}\left(n,n+1\right)\right)={\varPsi }^{n+1}{{\exp }}\left(i{k}_{3,n+1}{x}_{3}\left(n,n+1\right)\right)$$

Where *x*_*3*_(*n*, *n*+1) is the position of the interface between the *n*^th^ and (*n*+1)^th^ layers, and by the Born–von Karman condition for the whole stack,

3 $${\varPsi }^{1}{{\exp }}\left(i{k}_{3,1}{x}_{3b}\right)={\varPsi }^{3}{{\exp }}\left(i({k}_{3,3}{x}_{3t}+\varphi )\right)$$

where *x*_*3* _= *x*_*3t*_ and *x*_*3* _= *x*_*3b*_ are two planes of top and bottom, delimiting the volume of the trilayer stack. In other words, they correspond to the position of the upper boundary of the upper CoFeSiB layer (*x*_*3t* _= *L*_*3,1* _+ *L*_*3,2* _+ *L*_*3,3*_) and to the position of the lower boundary of the lower Pt layer (*x*_*3b*_ *=* *0*). Here *φ* is an additional phase considering the cumulative effect of mechanisms such as the presence of ultrathin auxiliary films like seed or capping layers or phase shifts induced at each interface.

The phase in the argument of the exponential functions is defined to within a 2*qπ* additive constant (*q* is an arbitrary integer).

4 $${k}_{3,1}{L}_{3,1}+{k}_{3,2}{L}_{3,2}+{k}_{3,3}{L}_{3,3}=2q\pi +\varphi$$

The last expression coinciding with Supplementary Eq. (6) can be derived from the Heisenberg uncertainty principle, as is shown in Supplementary Note 3. This quantization formalism can be easily extended to a general case of *N* constituent layers, in which case Eqs. (3–4) should be rewritten in the following way.

5 $${\varPsi }^{1}{{\exp }}\left(i{k}_{3,1}{x}_{3b}\right)={\varPsi }^{N}{{\exp }}\left(i({k}_{3,N}{x}_{3t}+\varphi )\right)$$

6 $${k}_{3,1}{L}_{3,1}+{k}_{3,2}{L}_{3,2}+{k}_{3,3}{L}_{3,3}+\ldots +{k}_{3,n}{L}_{3,N}=2q\pi +\varphi$$

Regarding the spin polarization of the itinerant electrons, it is supposed that the magnetization is in an in-plane direction (the *x*_*1*_ axis). The main idea is to compare the overall energy of the system in two possible states: (a) the electron spin, initially oriented with the magnetization in the FM layer, i.e., along with *x*_*1*_, is maintained after crossing the NS, and its polarization remains along with *x*_*1*_ in the HM film, and (b) the electron spin polarization is lost, and its state in the HM layer is random. This means that in the former case, the anti-symmetric term, a bilinear form based on the Levi–Civita anti-symmetric tensor often referred to as the Rashba term, which accounts for the effective SOC contribution must be retained in the Hamiltonian^15^, whereas in the latter case, the anti-symmetric term disappears.

More specifically, the anti-symmetric Rashba term contributing directly to the overall Hamiltonian is given by the following mixed product ${H}_{R}=\alpha (\left[\vec{{\boldsymbol{\sigma }}}\times \vec{{\bf{p}}}\right]\cdot {\vec{{\boldsymbol{n}}}}_{{\boldsymbol{int}}})$; here $\vec{{\boldsymbol{\sigma }}}$ is the electron polarization, $\vec{{\bf{p}}}$ is its momentum, ${\vec{{\bf{n}}}}_{{\bf{int}}}$ is a vector normal to the interface, while *α* is a constant depending on the physicochemical nature of the interface. In our case, where the normal to the interface is along *x*_*3*_, ${\vec{{\bf{n}}}}_{{\bf{int}}}$ = ${\vec{{\bf{u}}}}_{{\bf{3}}}$ and the spin polarization is assumed to be imposed by the magnetization in the FM layer, i.e., it is along *x*_*1*_, $\vec{{\boldsymbol{\sigma }}}$ = ${\vec{{\bf{u}}}}_{{\bf{1}}}$. This means that the only momentum component which can appear in the Rashba term is *p*_*2*_ *= ħk*_*2*_, thus we obtain *H*_*R*_ = −*J*∙*k*_*2*_ with *J* = *αħ*.

The FM/NS/HM structure may be regarded as a spin polarizer/phase shifter/spin momentum locker. If the presence of the spacer maintains the energy of the spin-polarized state at a lower level than the energy of the un-polarized state, then DMI occurs. Hence, the energy difference *ΔE* is supposed to be equal to the absolute value of the DMI surface energy. The values of the momenta of individual electrons appear directly in the corresponding energy expressions for each constituent layer, namely,

7 $${{\hslash }}\omega ={E}_{n}+(1/2){{{\hslash }}}^{2}({k}_{1}^{2}+{k}_{2}^{2}+{k}_{3,n}^{2})/{m}_{n}$$

for the layer *n* without the Rashba energy term (*n* = 2, 3), where *E*_*n*_ is the lowest energy level and *m*_*n*_ is the effective mass of the electron and

8 $${{\hslash }}\omega ={E}_{n{\prime} }+(1/2){{{\hslash }}}^{2}({k}_{1}^{2}+{k}_{2}^{2}+{k}_{3,{n}^{{\prime} }}^{2})/{m}_{{n}^{{\prime} }}-J{k}_{2}$$

in the layer with the Rashba contribution (*ń* = 1). Further details of the suggested formalism can be seen in Supplementary Note 3.

**Fig. 4:**
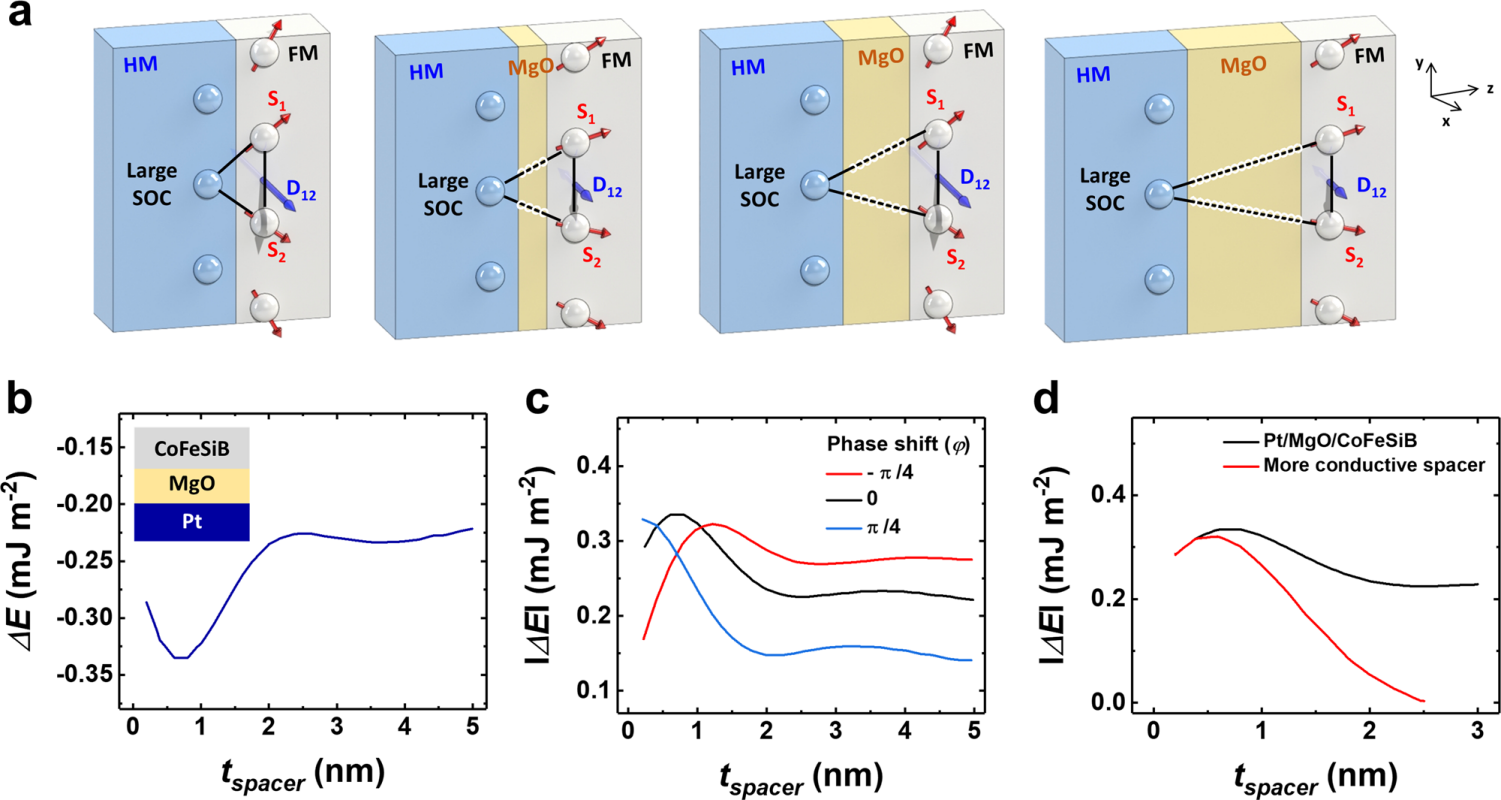
Theoretical calculations of the energy difference between the spin-polarized and non-polarized electron energy entering the HM layer. **a** Scheme of an iDMI at the interface between the HM (blue) and FM (gray). The iDMI vector (**D**_**12**_) is related to the triangle composed of two magnetic sites and an atom with a large SOC. The scheme represents the oscillating magnitude of **D**_**12**_ with corresponding spin tilting. **b** Energy difference (equal to the absolute value of the iDMI surface energy) calculated for the Pt/MgO/CoFeSiB system. **c** Shift of the oscillation-like behavior due to the assumption of a quantization condition Eq. (4) of ${k}_{3,1}{L}_{3,1}+{k}_{3,2}{L}_{3,2}+{k}_{3,3}{L}_{3,3}+\ldots =2\pi q+\varphi$ expressed in terms of the out-of-plane components of the circular wavenumbers *k*_*3,n*_ and thickness *L*_*3,n*_ of all three constituent layers (*n* = 1, 2, 3). The black curve represents *φ* = 0˚, while the red and blue curves represent *φ* = *π*/4 and *φ* = −*π*/4, respectively. The energy difference is displayed in terms of absolute value. d Theoretically calculated influence of the spacer nature on iDMI, where the black and red curve represents the energy difference for our sample (*E*_*F*_ –*E*_*MgO*_ = 9 × 10^−21^ J) and more conductive spacer sample (*E*_*F*_–*E*_*conductive spacer*_ = 12 × 10^−21^ J), respectively.

Figure 4a presents a schematic diagram of the oscillating magnitude of the iDMI vector depending on the MgO spacer thickness change. The results of our numerical simulations for the Pt/MgO(*t*_*MgO*_)/CoFeSiB configuration are shown in Fig. 4b. The curve (*ΔE(t*_*MgO*_*)*) reproduced the major trends in the experimental thickness dependence (*D*_*eff*_ (*t*_*MgO*_)) on a qualitative level. The reduction of the exchange energy was extremely slow, producing a characteristic pattern resembling oscillations (see Fig. 2c). As in the conventional RKKY, such oscillations typically result from the constructive or destructive interferences of the wave functions of itinerant electrons. The oscillating-like tendency in the theoretical simulations reproduces the major trend featured in the experiment, thus shifting the argument in favor of an oscillatory interpretation of the experimental curve in Fig. 2c. Intuitively, it is evident that the iDMI effective constant could decrease rather slowly under the assumption that the materials, including MgO in the stack, have similar free electron states. This implies the existence of an additional narrow band due to micro impurities and defects in MgO (see Supplementary Note 3).

Since the phase parameter *φ* directly appears in the law of quantization of the in-plane component of the circular wave number ${k}_{3}$ in Eq. (6), the oscillations featured by the theoretical curves could be shifted by varying $\varphi ,$ which provides us with an additional flexible instrument of fitting of numerical results to experimental data. Taking the parameters used for theoretical calculations (Supplementary Note 3) and *φ* = −*π*/4, 0, *π*/4, we obtained the phase-shifted energy difference curve (Fig. 4c). The phaseshifted the oscillations and changed the limit of iDMI energy per unit area. From this, the conflict between experimental and theoretical calculations of the phase difference could be resolved.

It would be instructive to draw a parallel with P. Bruno’s classical long-distance RKKY theory for IEC, suggesting a rapid exponential decay (complex Fermi surface) for dielectrics^45^ and 1/*x* with superimposed oscillations (conventional Fermi surface) for conducting metals^44^. Our theoretical analysis points out that MgO behaves rather like a conductor in the form of an extrinsic semiconductor. Consequently, as mentioned above, the *D*_*eff*_ (*t*_*MgO*_) dependence follows the oscillating 1/*x* pattern. The bandgap may vary with varying *t*_*MgO*,_ and there is no way to control it experimentally in ultrathin films like ours. Since these are the itinerant conduction electrons that are mainly involved in the spin transport, strictly speaking, this phenomenon cannot be regarded as conventional tunneling associated with the MgO spacers in TMR-related structures. For this reason, typically, in the text, we use the term, tunneling-like.

According to our theoretical analysis, based on preliminary numerical simulations, the observed abnormal transparency can occur only if the three constituent layers’ physical properties do not differ much. Although the electronic states of bulk Pt and CoFeSiB are clearly different, at both interfaces (Pt/MgO and MgO/CoFeSiB), where a strong hybridization is inevitable, the electronic properties of the active sublayers at the interfaces that are directly involved in the iDMI^28, 29^ can change considerably. This can lead to the creation of quite unusual states in these interfacial sublayers. In other words, it is fair to imagine that in the particular case of our samples, made of ultra-thin layers, probably the mixed electron states are very close in different constituents. Thus, in the Pt (5 nm)/MgO (0–2.1 nm)/CoFeSiB (0.9 nm) trilayer, the in-plane electron properties in the MgO film and both adjacent metallic layers, the Rashba-term carrying Pt and the Heisenberg-term carrying CoFeSiB, do not differ much. From the previous study, approximately four Pt monolayers (MLs) (*t*_*Pt*_ ~ 2 nm) are experimentally detected as active Pt sub layer^18^, and the CoFeSiB thickness of 0.9 nm corresponds exactly to three active MLs. Using the active thickness of Pt and CoFeSiB layer as parameters in the preliminary numerical simulations, we could obtain a maximum agreement between the theoretical and experimental curves. It may seem that our assumptions went too far, but they allow explaining the experimentally observed abnormal transparency of the MgO spacer.

Now let us consider a more conductive spacer (Fig. 4d) with a greater value of the energy range (*E*_*F*_ – *E*_*conductive spacer*_) than Pt/MgO/CoFeSiB case to discuss the influence of the spacer nature on iDMI. Based on the toy model, the more conductive spacer screens the iDMI. Although this appears somewhat counter-intuitive since the DMI is mediated by itinerant electrons in the context of the Fert-Levy model^11^, it was previously verified experimentally^20, 21^. The above numerical result only illustrates the general tendency of enhanced screening in heterogeneous structures with a more conductive spacer qualitatively. However, if we suppose that both interfacial metallic monolayers (Co and Pt) retain their electronic properties with respect to their bulk states, numerical simulations based on the toy model predict a very small tunneling distance of approximately 0.3 nm, which is in excellent quantitative agreement with the previously reported experimental data.

**Fig. 5:**
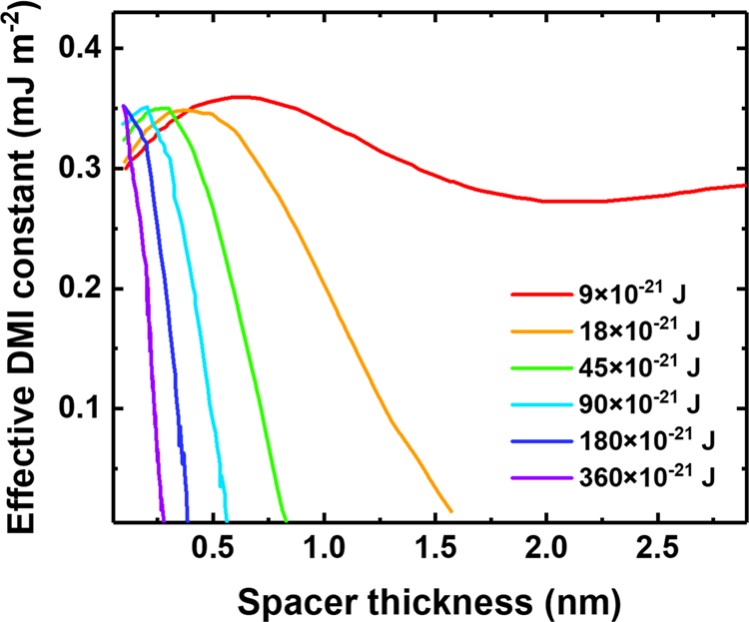
Theoretical estimation of the influence of the spacer energy range on iDMI. Each color represents a different *E*_*F*_ − *E*_*c,spacer*_ value. As the spacer becomes more conductive, the value of *E*_*F*_ − *E*_*c,spacer*_ increases. This approximate estimation is close to the energy range associated with a full screening with a spacer thickness equal to 0.3 nm, which is consistent with the experimental data for Co/Au/Pt stack^20^.

Our previous calculations in this paper have been performed for the following parameters: *m* = 18 × 10^−31^ kg and *E*_*F*_ – *E*_*c*_ = 9 × 10^−21^ J, *i.e*., 56 meV. These parameters correspond to a narrow band situation. To quantitatively estimate the influence of a good metal spacer, the energy range was varied, as shown in Fig. 5.

For a spacer conduction band filled in an energy range twice as large as the other ranges *E*_*F*_ – *E*_*c,spacer*_ = 18 × 10^−21^ J, the full screening is obtained with a thickness of 1.6 nm. For a spacer conduction energy range five times wider than the other layers, the full screening thickness falls to 0.8 nm. For a spacer energy range 10 times wider than the others, the full screening is observed with a thickness of 0.6 nm. Finally, the full screening with 0.3 nm is obtained with a spacer energy range equal 40 times larger than the other *E*_*F*_ – *E*_*c,spacer*_ = 360 × 10^−21^ J. The energy range for a good metal can be estimated as follows. The lattice parameter is about *α* ≈ 0.3 nm. The itinerant electron concentration is about *α*^−3^. This concentration corresponds to a Fermi vector **k**_**F**_ where $1/4{\pi }^{3}\times 4\pi /3\times {k}_{F}^{3}={a}^{-3}$, that is ${k}_{F}^{3}=3{\pi }^{2}{a}^{-3}$, i.e., *k*_*F*_ ≈ π*/α*. The corresponding energy range is ${E}_{F}-{E}_{c,spacer}={\hslash }^{2}{k}_{F}^{2}/2m={\hslash }^{2}{(\pi /a)}^{2}/2m=340\times {10}^{-21}J$. This approximate estimation is close to the energy range associated with a full screening with a spacer thickness equal to 0.3 nm. The calculations are consistent with the experimental data for Co/Au/Pt stack^20^.

The main equation governing the behavior of trilayer is the quantization condition (Eq. (6)), including all three layers’ contributions. In the case of a good metal as a spacer, the energy *E* – *E*_*c*_ is much larger than those in FM and HM; thus, the perpendicular wave vector $\sqrt{(2m(E-{E}_{c}))/{\hslash }^{2}-{k}_{//}^{2}}$ is also much larger. Consequently, the HM contribution containing the DMI related Rashba term becomes negligibly small and disappears from the quantization condition. Thus, iDMI is canceled.

We would like to emphasize that the aim of our calculations in the framework of our toy theory is not to achieve exact figures for any given system, which would be unrealistic at this stage, but to provide purely approximate estimations as well as to outline qualitative trends peculiar to the systems under investigation. Characteristically, it has succeeded in predicting the possibility of highly transparent spacers in a trilayer with similar physical properties observed in the present study and providing a qualitative and quantitative explanation for the alternative entirely non-transparent behavior of metallic spacers observed experimentally in earlier studies.

Given the complexity of the problem, only a combined approach based on innovative and clear analytics, providing for physical insight, and ab initio numerical simulations allowing checking the validity of major assumptions can succeed in a truly comprehensive theoretical analysis.

## Discussion

We have addressed a fundamental issue of the role of a non-metallic MgO spacer as a conveyor of iDMI between FM and HM layers and the physics behind it. We report the results of the direct experimental investigation of the evolution of the strength of iDMI as a function of the thickness of the MgO spacer (0 < *t*_*MgO*_ < 2.1 nm), which has revealed non-trivial features. Thus, it has turned out that the experimentally observed phenomenon cannot be regarded as conventional tunneling associated with the MgO spacers in TMR-related structures. In the case of an isolator spacer such as MgO, the conventional behavior is characterized by an abrupt exponential decrease of the inter-layer exchange RKKY coupling. Instead, the iDMI coupling drops down slowly in a non-monotonic fashion resembling the oscillations in the case of RKKY coupling of two FM films through a nonmagnetic conducting spacer. To explain the experimental results and to identify the potential physical mechanisms behind the unconventional magnetic behavior of the investigated system, an original ad hoc analytic formalism, relying on general energy-related terms, has been developed. It can explain the high transparency of the MgO spacers under investigation. Our theoretical analysis points out that MgO seems to behave like a conductor in the form of an extrinsic semiconductor. Due to the oxygen deficiency, an impurity sub-band was formed below the conduction band, enabling the MgO spacer to possess electronic states similar to those in the adjacent sublayers in Pt and CoFeSiB. Moreover, this approach can show both qualitative and quantitative pathways for alternative entirely non-transparent behavior of metallic spacers observed experimentally in earlier studies. To conclude, the results reported in this paper, experimental and theoretical, show clues for new mechanisms allowing tuning the magnitude of iDMI by engineering the spacer layer thickness between the HM and FM layers, providing a powerful tool for realizing the skyrmion-based memory, logic, and neuromorphic devices.

## Methods

### Sample preparation

Samples were deposited on 1.25 × 1.25 cm^2^ thermally oxidized Si wafers using an ultra-high vacuum magnetron sputtering system with a base pressure of 5 × 10^−9^ Torr. The stacking structure of the sample is Ta(3)/Pt(5)/MgO(*t*_*MgO*_)/CoFeSiB(0.9)/MgO(1)/Ta(2) on Si substrates with thermal oxides, where *t*_*MgO*_ = 0–2.1 nm. The 2-nm-thick Ta buffer layer below Pt was used to enhance the crystal structure characteristics of the Pt layers and induce a better interface between the HM and FM layer^49^. Pt is a promising candidate in spintronics because it provides the highest value of the iDMI constant. The MgO thickness between Pt and CoFeSiB was varied to identify the effect of the spacer layer between Pt and CoFeSiB on the magnetic properties and iDMI energy density. The composition of CoFeSiB is Co_70.5_Fe_10.5_Si_15_B_10_ in at%. The structural symmetry breaking-induced iDMI effect was excluded when MgO was inserted between HM and FM: (1) MgO was deposited on top of CoFeSiB, and (2) Ta functioned as an oxidation protection layer. DC and RF sputtering guns were used to deposit the metal and oxide layers, respectively.

### Magnetic and microstructural characterizations

The hysteresis loops of the as-deposited samples were measured using a vibrating sample magnetometer (VSM, Microsense EV9). The magnitude and sign of iDMI were probed by Brillouin light scattering (BLS) spectroscopy. The frequency shift of the inelastically scattered light was analyzed using a tandem 2 × 3 pass Sandercock Fabry-Pérot interferometer, which typically allowed access to a 3–300 GHz spectral frequency range. We used the Damon-Eshbach geometry with the optical angle of incidence typically equal to 60°. Thus far, this technique has been proven to be the most direct and reliable for measuring the effective value of the intrinsic iDMI constant. The XRD measurement of 2*θ*–*ω* scanning was taken with the incident angle ranging from 30 to 80° at 0.02° steps. A tilted angle of 5° was used to prevent the signal arising from the Si substrate. The microstructural analysis was conducted, and the crystal structure of Pt and MgO spacer layer using a transmission electron microscope (TEM, Titan TM 80-300, FEI Company) and x-ray diffraction (PANalytical, X’Pert ProMPD) with Cu K$a$ radiation (*λ* = 1.5406 Å). The elemental depth profiles of the films were determined by SIMS (ION-TOF, TOF.SIMS 5) using Cs^+^ ions. After sputtering sequence with an incident current of 0.5 keV and 5 nA on 300 × 300 μm^2^ unit area, the analysis was done with a current of 30 keV and 1 pA on 100 × 100 μm^2^. The bandgap energy of MgO with an incident energy of 1 keV was measured using REELS (Axis-Supra, Kratos).

## Supplementary information

Supplementary Information

## Data Availability

The data that support the findings of this study are available from the corresponding author upon reasonable request.
