# Supplementary material for: Ruderman–Kittel–Kasuya–Yosida-type interfacial Dzyaloshinskii–Moriya interaction in heavy metal/ferromagnet heterostructures
Source: Nat Commun. 2021 Jun 2;12:3280. doi: 10.1038/s41467-021-23586-y (PMC8172855; doi:10.1038/s41467-021-23586-y)
Supplement: Supplementary file 1 — Supplementary Information [file 41467_2021_23586_MOESM1_ESM.pdf]

Supplementary Information

Ruderman-Kittel-Kasuya-Yosida-type interfacial Dzyaloshinskii-Moriya interaction in heavy metal/ferromagnet heterostructures

Taehyun Kim[1], In Ho Cha[1], Yong Jin Kim[1], Gyu Won Kim[1], Andrey Stashkevich[2], Yves Roussigné[2], Mohamed Belmeguenai[2], Salim M. Chérif[2], Alexander Samardak[3,4], Young Keun Kim[1*]

[1]Department of Materials Science and Engineering, Korea University, Seoul 02841, Korea

[2]Laboratoire des Sciences des Procédés et des Matériaux, CNRS-UPR 3407, Université Sorbonne Paris Nord, Villetaneuse, 93430 France.

[3]School of Natural Sciences, Far Eastern Federal University, Vladivostok 690950, Russia

[4]National Research South Ural State University, Chelyabinsk 454080, Russia

*e-mail: ykim97@korea.ac.kr (Y. K. Kim)

Supplementary Note 1. Uniaxial anisotropy energy

The behavior of $K_{u,eff}$ for t_{FM} ranging from 0.6 to 1.2 nm can be fitted with a negatively sloping line: the slope indicates volume anisotropy, whereas the intercept with the vertical axis provides the surface anisotropy. The obtained gradients for the volume anisotropy correspond to $-7.5\times10^{6} \pm 2.2\times10^{6}$ and $-6.2\times10^{6} \pm 1.0\times10^{6}$ mJ m^{-2} for 0 and 1.2 nm t_{MgO} samples, respectively. From the relationship of the effective energy of magnetic anisotropy, the volume contribution is given by $K_v = K_{sh} + K_{mc} + K_{me}$, where the dipole-induced shape anisotropy $K_{sh} = -2\pi\left(M_s^{eff}\right)^2$, and K_{mc} and K_{me} are the magnetocrystalline and magnetoelastic contributions, respectively. From the calculation, the shape anisotropy energies are -6.2×10^{6} and -5.1×10^{6} mJ m^{-2} for 0 and 1.2 nm of t_{MgO} samples, respectively. These values are close to those of the volume anisotropy, suggesting that the contributions of K_{mc} and K_{me} are negligible. From $K_s = (K_{eff} + 2\pi M_s^2)$, the contribution of K_s for 0 and 1.2 nm of MgO with t_{FM} = 0.9 nm are 0.48 and 0.43 mJ m^{-2}, respectively.

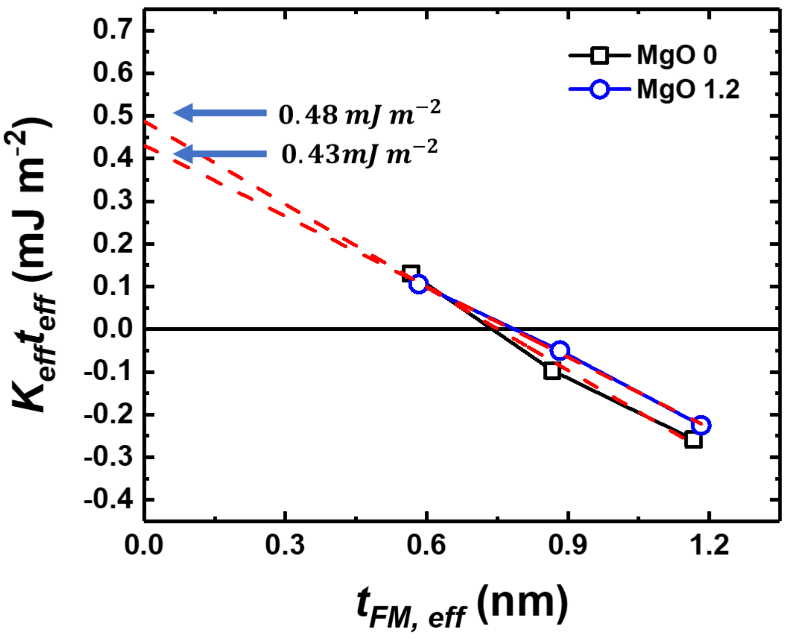

Supplementary Fig. 1 Dependence of $K_{eff}t_{eff}$ on $t_{FM,eff}$ for Ta/Pt/MgO/CoFeSiB($t_{FM,eff}$)/MgO/Ta samples, where t_{MgO} = 0 and 1.2 nm.

Supplementary Note 2. Details of Brillouin Light Scattering measurement

The interfacial Dzyaloshinskii-Moriya interaction (IDMI) is characterized by the same symmetry as the Landau-Lifshitz equation. Consequently, the presence of IDMI modifies the conventional Damon-Eshbach (DE) dispersion relation in a characteristic straightforward manner, namely, the IDMI contribution to the spin-wave (SW) frequency appears as a linear addition that scales as the SW wavenumber k_{sw}[1]. In terms of wave science, this corresponds to the introduction of linear dispersion, producing a non-reciprocity in the SW propagation. In other words, two counters propagating DE SWs with the same k_{sw} will have different frequencies. This frequency difference (Δf) will be proportional to k_{sw} due to the non-reciprocity of the Stokes (Doppler down-shifted magneto-optical (MO) response with the peak frequency f_S) and anti-Stokes (Doppler up-shifted MO response with the peak frequency f_{AS}) SW propagation[2]. Stokes and anti-Stokes peaks are shown in Supplementary Figure 2a, b.

$$\Delta f = f_S - f_{AS} = \frac{2\gamma}{\pi}\frac{D_{eff}}{M_S}k_{sw} \tag{1}$$

Here M_s is the saturation magnetization of the ferromagnetic layer, γ is the gyromagnetic ratio $\frac{\gamma}{2\pi} = g \times 13.996$ GHz/T, where g is the Landé factor), and D_{eff} is the effective iDMI at a particular NM/FM interface. The BLS in the backscattering configuration, in which case a straightforward relation between k_{sw} and the angle of incidence θ of the optical beam with a wavelength λ_{opt} , $k_{sw} = (4\pi \sin(\theta))/\lambda_{opt}$ exists, is ideally suited for this kind of measurements.

Special measures were undertaken to ensure reliable Stokes/anti-Stokes frequency asymmetry detection both in the experimental procedure and in the processing of the BLS spectra. To minimize the instrumental error and to improve the quality of the raw BLS data, spectra were typically obtained after counting photons for 12 hours. To validate our results, we utilize the fact that the sign of IDMI is changed if the saturating magnetic field polarity is

inversed[3]. The Stokes f_S and anti-Stokes f_{AS} frequencies were determined from Lorentzian fit to the spectral peaks. Moreover, we took the BLS spectra without an analyzer, thus recording not only the peaks corresponding to light scattering by magnons but also those representing phonons. Notably, the propagation of acoustic phonons is never non-reciprocal, making them very effective for checking the calibration of the frequency sweeping of the BLS set-up.

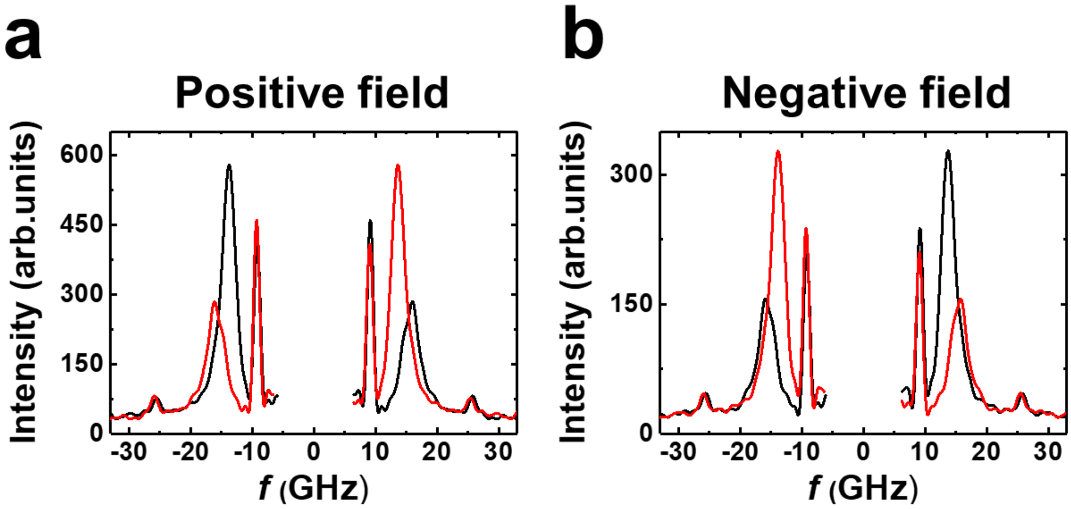

Supplementary Fig. 2 a, b The panels detail smoothed spectrums (10-point filtering, black line). The red line is its mirror reflection with respect to the vertical axis to make the Stokes/anti-Stokes asymmetry more visible.

To minimize the risk of any misinterpretation of the experimental results, comprehensive numerical modeling of the BLS spectral lines has been carried out. It is a most general formalism based on the fluctuation-dissipation theorem and optical Green's functions to consider the optical properties of the structure developed. Thus, both the thermal stochastic nature of the magnons and the magneto-optical properties of the structure were simultaneously considered.

Supplementary Note 3. Theoretical Calculations

As mentioned in the main text, the free electron model plays a vital role in our theoretical analysis. A free-electron state with an energy $\hbar\omega$ and a momentum $\vec{\boldsymbol{p}} = \hbar\vec{\boldsymbol{k}}$ is associated with a plane wave and the expression of the corresponding wave function reads,

$$\Psi = \Psi^{o}\exp\left(i(k_1x_1 + k_2x_2 + k_3x_3 - \omega t)\right) \quad (2)$$

In the non-relativistic limit, we have $\hbar\omega = \hbar^2(k_1^2 + k_2^2 + k_3^2)/2m$ where m is the electron mass. For an electron moving in a non-vacuum medium, the electron mass must be replaced with its effective mass. If the electron is confined within a box with dimensions $L_1 \times L_2 \times L_3$, the momentum components have quantized values defined by the relation identical to that of the transverse resonance in classical optics or acoustics, stating that the difference between two nearest permitted values of the angular wave number δk (increment of the angular wave number or the quantization step) corresponds to an additional phase shift of 2π.

$$\delta k_1L_1 = \delta k_2L_2 = \delta k_3L_3 = 2\pi \quad (3a)$$

This relation may be regarded as a consequence of the Heisenberg uncertainty principle in quantum mechanics establishing the relation between the uncertainty in the definition of the particle's momentum ($\delta p = \hbar\delta k$) and its position (δx). In this regard, the uncertainties δk and δp can be identified with the increment of the mean angular wave number and mean momentum, correspondingly, while the position uncertainty with the corresponding size $L_1 \times L_2 \times L_3$ of the layer. Thus Supplementary Eq. (3a) rewritten correspondingly coincides with the conventional position-momentum uncertainty relation.

$$\hbar\delta k_1L_1 = \hbar\delta k_2L_2 = \hbar\delta k_3L_3 = h \quad (3b)$$

For a layer in which L_1 and L_2 are very large relative to L_3, according to Supplementary Eq. (3a), the quantization steps δk_1 and δk_2 are vanishingly small $\delta k_1 << \delta k_3$; $\delta k_2 << \delta k_3$ and consequently, we can assume that k_1 and k_2 form a continuum, whereas k_3 takes discrete values $k_3 = q\delta k_3$ where q is an integer.

Let us now consider a multilayer comprised of N different media. As before, according to the uncertainty principle, the mean momentum increment times the position uncertainty (layer thickness) is equal to the Planck's constant:

$$\hbar\delta k_{3,1}L_{3,1} + \hbar\delta k_{3,2}L_{3,2} + \hbar\delta k_{3,3}L_{3,3} + \cdots + \hbar\delta k_{3,N}L_{3,N} = h \qquad (4)$$

where $L_{3,1}$, $L_{3,2}$, $L_{3,3}$, … are the thicknesses of each layer. From this relation, one obtains

$$\hbar k_{3,1}L_{3,1} + \hbar k_{3,2}L_{3,2} + \hbar k_{3,3}L_{3,3} + \cdots + \hbar k_{3,N}L_{3,N} = qh + \hbar\varphi \qquad (5)$$

φ is a constant discussed below. Supplementary Eq. (5) can be rewritten in its final form, coinciding with Eq. (6) in the main text.

$$k_{3,1}L_{3,1} + k_{3,2}L_{3,2} + k_{3,3}L_{3,3} + \cdots + k_{3,N}L_{3,N} = 2q\pi + \varphi \qquad (6)$$

An additional phase parameter φ is introduced to consider possible phase shifts whose origin is discussed in the main text.

In our case of the investigation of the Pt/MgO/CoFeSiB tri-layer, a particular example of an FM/NS/HM structure, CoFeSiB film plays the role of the polarizer of itinerant electrons within which electron spins are aligned with the magnetization through s-d conventional exchange interaction. This direction is supposed to be parallel to the x_1 axis. On the other hand, the permitted quantized values of the out-of-plane components of the circular wavenumbers $k_{3,n}$ in each layer n are given by Supplementary Eq. (6) (with $N = 3$) (see also Eq. (4) in the main text) as well as the corresponding quantized energy levels in CoFeSiB, MgO, and Pt are described by the expression below.

$$\hbar\omega = E_{CoFeSiB} + \hbar^2(k_1^2 + k_2^2 + k_3^2)/(2m_{CoFeSiB}) \qquad (7)$$

$$\hbar\omega = E_{MgO} + \hbar^2(k_1^2 + k_2^2 + k_3^2)/(2m_{MgO}) \qquad (8)$$

$$\hbar\omega = E_{Pt} + \hbar^2(k_1^2 + k_2^2 + k_3^2)/(2m_{Pt}) - Jk_2 \qquad (9)$$

Here, $E_{CoFeSiB}$, E_{MgO}, and E_{Pt} are the lowest energy levels while $m_{CoFeSiB}$, m_{MgO}, and m_{Pt} are effective masses of itinerant electrons, correspondingly, in CoFeSiB, MgO, and Pt.

In Pt, a spin-orbit coupling-related DMI contribution characterized by the Rashba term

(antisymmetric exchange) appears in the energy. Its structure is explained in the main text. The total energy per unit area is the sum of all the quantized energies weighted by their probability.

$$E_{total} = \iint \left(\frac{dk_1}{2\pi}\right)\left(\frac{dk_2}{2\pi}\right) \sum \hbar\, \omega_q / (1 + exp\, (\frac{(\hbar\omega_q - E_F)}{k_B T}) \qquad (10)$$

The quantized energy levels $\hbar\omega_q$ in S(10) labeled by q are given by Supplementary Eq. (6-9). Two alternative energy expressions will be derived: we assume that the electrons either retain their well-defined spin polarization or lose their spin polarization after crossing the spacer. In the latter case, the spin-orbit coupling contribution, parametrized by the constant *J,* is set to zero. If the energy corresponding to the former case is lower than the energy corresponding to the latter case, DMI coupling is possible. The energy difference is interpreted as the absolute magnitude of the DMI energy per unit area.

Our main experimental result consists of the non-trivial fact that a MgO spacer remains practically iDMI transparent for relatively large thicknesses (of up to 2.1 nm). Preliminary numerical simulations have revealed that such feature is possible if the physical parameters of all the three constituent layers tend to similar values. In this regard, it would be interesting to assume that Pt and CoFeSiB present similar electronic states; we can take $E_{Pt} = E_{CoFeSiB}$ and $m_{Pt} = m_{CoFeSiB}$. Moreover, as MgO in our sample seems to be transparent, we assume that because of defects, some states exist in MgO, which are similar to those in CoFeSiB.

Thus, we take $E_{CoFeSiB} = E_{MgO}$ and $m_{CoFeSiB} = m_{MgO}$. Using $L_{CoFeSiB}$ = 1 nm, L_{Pt} = 2 nm, $E_F - E_{CoFeSiB} = E_F - E_{Pt} = E_F - E_{MgO} = 9 \times 10^{-21}$ J, $m_{Pt} = m_{CoFeSiB} = m_{MgO} = 18 \times 10^{-31}$ kg and $J = 3 \times 10^{-29}$ J m. Such assumptions have allowed us to reproduce the major features of the experimental curve, namely, a slow non-monotonic decrease of the DMI energy per unit area with some resemblance to oscillations (Fig. 4b). One can find more details on the results of the numerical simulations in the main text.

Supplementary Note 4. Scanning Transmission Electron Microscope (STEM) image

Supplementary Figure 3 represents scanning transmission electron microscope (STEM) image and energy dispersive X-ray spectroscopy (EDX)-line profile of the sample studied. From the STEM image and EDX-line profile, a clear multilayer of the entire stack is observed. The specific crystal structure of Pt is analyzed in the main text.

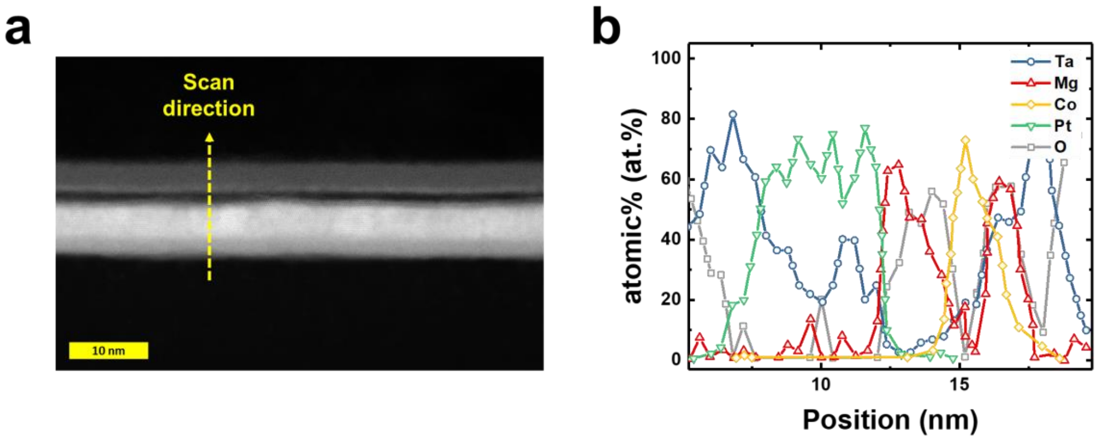

Supplementary Fig. 3 Structure of the Ta 3/Pt 5/MgO 2/CoFeSiB 0.9/MgO 1 (in nm). a STEM image of the entire structure under study. **b** EDX-line profile with scanning direction indicated in Supplementary Figure 3a.

Supplementary Note 5. Secondary ion mass spectroscopy (SIMS) elemental depth profile

We performed secondary ion mass spectroscopy (SIMS) elemental depth profiling to further analyze the sandwich structure studied. Supplementary Figures 4a and b show the elemental distributions in the samples with and without the MgO spacer. The SIMS depth profiling analysis supported that the structure we used for BLS measurement was deposited with the expected stacking sequence. The diffusion of Co through MgO was not observed from the SIMS depth profile. Since the sample we studied did not undergo any thermal treatment, diffusion of magnetic material is not expected.

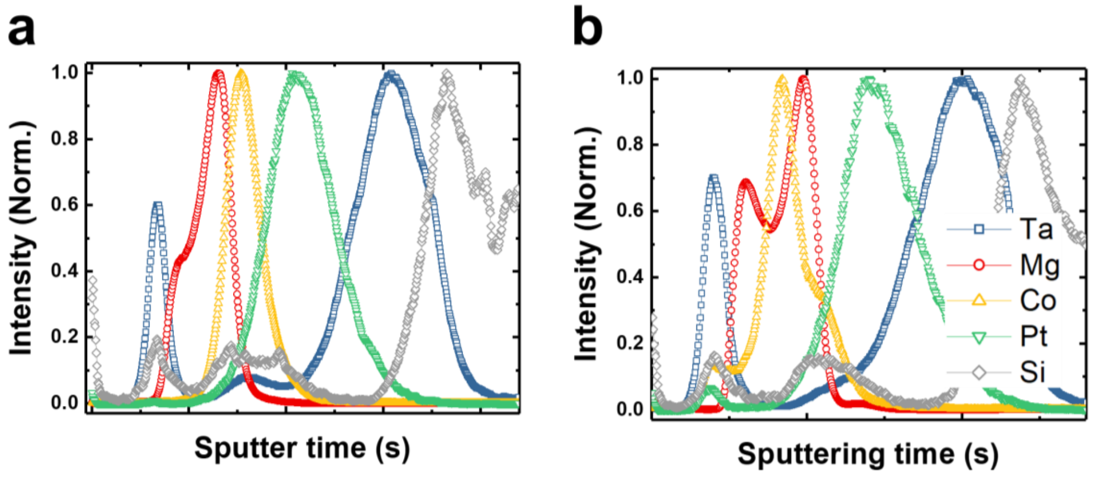

Supplementary Fig. 4 Secondary ion mass spectroscopy (SIMS) elemental depth profile of Ta(3)/Pt(5)/MgO(t_{MgO})/CoFeSiB(0.9)/MgO(1)/Ta(2). a, b SIMS depth profiles for t_{MgO}=0 (**a**) and t_{MgO}=2.1 nm (**b**) films, respectively.

Supplementary References

1. Kostylev, M. Interface boundary conditions for dynamic magnetization and spin wave dynamics in a ferromagnetic layer with the interface Dzyaloshinskii-Moriya interaction. *J. Appl. Phys.* **115**, 233902 (2014).
2. Belmeguenai, M., Gabor, M. S., Roussigné, Y., Stashkevich, A., Chérif, S. M. *et al.* Brillouin light scattering investigation of the thickness dependence of Dzyaloshinskii-Moriya interaction in $Co_{0.5}Fe_{0.5}$ ultrathin films. *Phys. Rev. B* **93**, 174407 (2016).
3. Stashkevich, A. A., Belmeguenai, M., Roussigné, Y., Cherif, S. M., Kostylev, M. *et al.* Experimental study of spin-wave dispersion in Py/Pt film structures in the presence of an interface Dzyaloshinskii-Moriya interaction. *Phys. Rev. B* **91**, 214409 (2015).
